# Enhanced Chondrogenic Potential and Osteoarthritis Treatment Using Cyaonoside A-Induced MSC Delivered *via* a Hyaluronic Acid-Based Hydrogel System

**DOI:** 10.14336/AD.2024.10016

**Published:** 2026-01-29

**Authors:** Xingyan An, Qirong Zhou, Shihao Sheng, Anfu Deng, Han Liu, Xiuhui Wang, Qin Zhang, Yingying Jing, Ke Xu, Chongru He, Robert Chunhua Zhao, Jiacan Su

**Affiliations:** ^1^Institute of Translational Medicine, Shanghai University, Shanghai, China.; ^2^Organoid Research Center, Shanghai University, Shanghai, China.; ^3^Institute of Basic Medical Sciences Chinese Academy of Medical Sciences, School of Basic Medicine Peking Union Medical College, Beijing, China.; ^4^Center for Excellence in Tissue Engineering, Chinese Academy of Medical Sciences, Beijing Key Laboratory of New Drug Development and Clinical Trial of Stem Cell Therapy, Beijing, China.; ^5^School of Life Sciences, Shanghai University, Shanghai, China.; ^6^Department of Orthopaedics, Xinhua Hospital, Shanghai Jiao Tong University School of Medicine, Shanghai, China.; ^7^Sanming Institute of Translational Medicine, Sanming, China.; ^8^Wenzhou Institute of Shanghai University, Wenzhou, China.; ^9^Wenzhou Key Laboratory of Tissue Regeneration Medical Materials, Wenzhou, China.

**Keywords:** Cyaonoside A, MSC, knee osteoarthritis, hydrogel delivery system

## Abstract

Osteoarthritis (OA) is a prevalent degenerative joint disease that significantly impacts the quality of life in the elderly. Traditional Chinese medicine, particularly Medicinal Cyathula Root and its active component Cyaonoside A (CyA), has been utilized to treat OA by promoting chondrocyte proliferation, inhibiting inflammatory factors, and maintaining joint homeostasis. Concurrently, mesenchymal stem cells (MSC) derived from placental umbilical cord, bone marrow, and adipose tissue have gained attention for their potential in OA treatment due to their chondrogenic differentiation capabilities. This study explored the therapeutic synergy of CyA and MSC for enhanced cartilage regeneration. Optimal chondrogenic differentiation was achieved by treating MSC with 0.5 mg/mL CyA for 3 days, significantly increasing the expression of key cartilage-specific genes ACAN, COL2A, and SOX9. Comparative gene expression and pathway analyses revealed that CyA-induced MSC (C-MSC) modulate critical signaling pathways, including TGF-β, PI3K-Akt, and Wnt, demonstrating their potential in cartilage repair. Furthermore, C-MSC-derived exosomes exhibited superior anti-inflammatory and anti-apoptotic effects compared to MSC-derived exosomes in IL-1β-treated human chondrocytes, enhancing chondrogenic gene expression and reducing cartilage degradation. To enable targeted delivery, a novel injectable hydrogel system (HAMA@C-MSC) was developed using methylacrylated hyaluronic acid (HAMA). This hydrogel facilitated uniform cell distribution, maintained structural integrity, and demonstrated excellent biocompatibility and biosafety, with no cytotoxic or hemolytic effects. In vivo studies using a rat destabilization of the medial meniscus OA model confirmed that HAMA@C-MSC significantly improved cartilage structure, enhanced chondrocyte regeneration, and restored collagen integrity, outperforming other treatment groups as validated through imaging, histology, and molecular analyses. These findings highlight HAMA@C-MSC as a promising therapeutic strategy for OA, leveraging the synergistic effects of C-MSC and advanced hydrogel technology to achieve enhanced cartilage regeneration and joint protection.

## INTRODUCTION

Osteoarthritis (OA) is one of the most common diseases globally, notably diminishing life quality for individuals over 40 [1]. Factors such as trauma, aging, excess weight, and climatic exposure, alongside decreased estrogen in postmenopausal women, contribute to its development. The disease progressively damages articular cartilage, leading to severe pain, reduced mobility, and potentially, disability or death. Current treatments, categorized into early and late stages, range from pharmacological and rehabilitative approaches to total knee replacement, yet none can regenerate damaged cartilage, underscoring the urgent need for innovative solutions.

Stem cells have demonstrated significant potential in treating OA due to their remarkable regenerative properties. However, their targeting capabilities remain suboptimal. Previous research has established that Cyanoside A significantly inhibits the production of inflammatory factors, protects chondrocytes, and enhances the regeneration of articular cartilage [2]. Further, it has been shown that a controlled dose of Cyanoside A can effectively promote the differentiation of stem cells into chondrocytes over a specific period [3]. Nevertheless, excessive doses or prolonged treatment can inhibit this effect, likely due to the dose-dependent toxicity associated with the monomers of traditional Chinese medicine [4]. Additionally, it has been observed that exogenous medications can influence the therapeutic effects of stem cells by altering their differentiation potential. Notably, compared to natural mesenchymal stem cells (MSC), those induced by medication exhibit enhanced paracrine capabilities and anti-inflammatory effects [5, 6].

**Figure 1.**
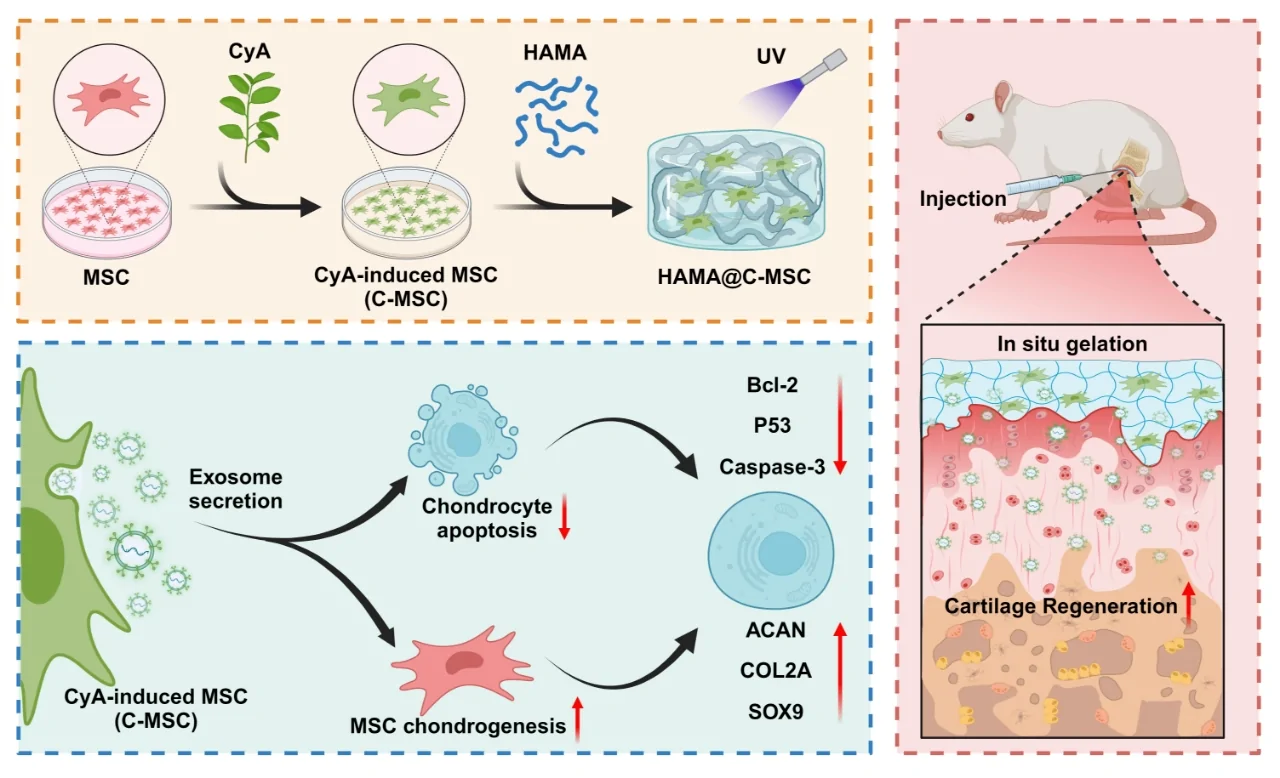
Structure and Efficacy of the HAMA@C-MSC Delivery System for Treating Knee Osteoarthritis.

In this study, a novel type of MSC, named Cyaonoside A-induced mesenchymal stem cells (C-MSC), was developed by inducing traditional MSC with the Chinese medicine monomer Cyanoside A [7, 8]. The optimal concentration and timing for this induction were identified. Transcriptome sequencing analysis and subsequent verification of the chondrogenic differentiation mechanism revealed that C-MSC exhibit enhanced chondrogenic potential compared to traditional MSC, suggesting a superior efficacy in treating OA. The study further explored the specific regulatory mechanisms through which C-MSC promotes chondrogenic differentiation of MSC and inhibits cartilage cell damage. Emphasis was placed on their ability to reduce inflammatory reactions, improve cartilage cell functionality, and decrease apoptosis, thereby confirming the significant potential of these monomer-induced stem cells in OA treatment. Additionally, to maximize therapeutic outcomes, C-MSC were incorporated into a hydrogel, enhancing their sustained effectiveness and impact within the joint cavity (Fig. 1). The findings underscore the broad therapeutic prospects of C-MSC in OA treatment.

## MATERIALS AND METHODS

### Materials and cells

Cyaonoside A (CyA) was purchased from Chengdu HerbSubstance Co., Ltd. (China). IL-1β was acquired from MedChemExpress (MCE, USA). Human chondrocytes and mesenchymal stem cells (MSC) were obtained from Cyagen Biosciences Inc. (China). The culture medium, fetal bovine serum (FBS), and penicillin/streptomycin (PS) were sourced from HyClone (USA). Hyaluronic acid methacryloyl (HAMA) was procured from Engineering For Life (EFL, China).

All cells were cultured in a suitable medium containing 10% FBS and 100 U/mL PS. The cells were incubated at 37° with 5% CO2 and saturated humidity in a thermostatic incubator. The medium was changed every 48 hours. Cells were digested and passaged upon reaching 80% confluence. CyA-induced MSC (C-MSC) were obtained by treating adherent MSC with 0.5 mg/mL CyA for three days. All other reagents were purchased from Macklin (China), unless otherwise noted.

### Preparation and characterization of HAMA@C-MSC

Dissolve HAMA in a phosphate-buffered saline (PBS) solution containing 0.25% photoinitiator LAP (EFL, China) to prepare a 5% hydrogel precursor solution. To prepare HAMA@C-MSC hydrogels, 5 million C-MSC were mixed with 500 μL of the hydrogel precursor solution. The injectability of HAMA@C-MSC was assessed using a sterile syringe and documented through photography before UV cross-linking. Under the irradiation of 405 nm UV light, the HAMA@C-MSC hydrogel rapidly solidified.

After freeze-drying, the internal microscopic morphology of the hydrogel was examined using a scanning electron microscope (SEM, Hitachi, Japan). The storage modulus (G') and loss modulus (G'') of both HAMA and HAMA@C-MSC were measured using a rheometer (TA Instruments, USA). The compression modulus of all samples was determined with a universal mechanical testing machine (Shimadzu, Japan). The weight of the hydrogel after soaking in PBS was recorded at predetermined intervals to calculate the swelling rate. To determine the degradation rate, HAMA and HAMA@C-MSC were incubated in PBS containing 10 U/mL hyaluronidase at 37°C and 100 rpm in a constant temperature shaker.

The degradation rate (%) was calculated using the formula: Degradation rate (%) = (W_0_-Wc)/W0×100, where W_0_ represents initial weight, and Wc represents current weight.

### Extraction and characterization of exosomes

To isolate exosomes, cell culture supernatants were first transferred to new centrifuge tubes and centrifuged at 2,000 × g for 30 minutes at 4 °C to pellet cell debris. The supernatant was then carefully transferred to a fresh centrifuge tube and centrifuged at 10,000 × g for 45 minutes at 4 °C to remove larger vesicles. The cleared supernatant was subsequently filtered through a 0.45 μm filter membrane (Millipore, USA) to exclude smaller particulates. This filtrate was then transferred to another new centrifuge tube and ultracentrifuged at 100,000 × g for 70 minutes at 4 °C using an appropriate rotor (Hitachi, Japan). The supernatant was discarded, and the pellet was resuspended in 10 mL of pre-cooled PBS. This suspension was ultracentrifuged again under the same conditions. After discarding the final supernatant, the pellet was resuspended in 200 μL of pre-cooled PBS and stored at -80 °C for long-term preservation.

For TEM analysis, 10 μL of the exosome suspension was placed onto a formvar-coated copper grid and allowed to settle for 1 minute. Excess fluid was removed with filter paper. The sample was then negatively stained with 10 μL of uranyl acetate for 1 minute, and excess stain was absorbed using filter paper. The grids were allowed to dry at room temperature for several minutes before imaging with a transmission electron microscope (TEM, Hitachi, Japan) at 100 kV. For nanoparticle tracking analysis, the exosome preparation was diluted in PBS and analyzed immediately using a Particle Metrix system (Germany).

### Alcian Blue staining

Alcian Blue, a cationic dye, reacts with the acidic proteoglycans in the extracellular matrix of cartilage, producing a blue color, and is thus used to assess chondrocyte differentiation and normal extracellular matrix synthesis. To perform staining, adherent cells cultured in 24-well plates were removed from the medium and rinsed once with PBS. Cells were then fixed with paraformaldehyde (specific concentration needed, e.g., 4%) for 15 minutes at room temperature. Alcian Blue staining solution (Beyotime, China) was subsequently added for 20 minutes. After staining, any residual dye was thoroughly washed away with ultrapure water. Photographs of the stained cells were taken to document the results once the wells were clear of any unbound dye.

### RNA-seq sequencing

Total RNA was isolated using the Trizol Reagent (Invitrogen, USA). The concentration, quality, and integrity of the RNA were assessed using a NanoDrop spectrophotometer (Thermo Scientific, USA). Three micrograms of RNA were used as input material for the RNA sample preparations. The sequencing libraries were generated through several steps. First, mRNA was purified from total RNA using poly-T oligo-attached magnetic beads. Fragmentation was performed using divalent cations at elevated temperatures in an Illumina proprietary fragmentation buffer. First-strand cDNA synthesis was conducted using random oligonucleotides and SuperScript II. This was followed by second-strand cDNA synthesis using DNA Polymerase I and RNase H. Remaining overhangs were converted to blunt ends via exonuclease and polymerase activities, after which the enzymes were removed. After adenylation of the 3' ends of the DNA fragments, Illumina PE adapter oligonucleotides were ligated to prepare for hybridization. To select cDNA fragments of the preferred 400-500 bp length, the library fragments were purified using the AMPure XP system (Beckman Coulter, USA). DNA fragments with ligated adaptor molecules on both ends were selectively enriched using the Illumina PCR Primer Cocktail in a 15-cycle PCR reaction. The products were purified and quantified using the Agilent High Sensitivity DNA assay on a Bioanalyzer 2100 system (Agilent, USA). Finally, the sequencing library was sequenced and analyzed on the NovaSeq 6000 platform (Illumina, USA).

### Cell Viability Assay

The CCK-8 assay was used to assess cell viability across different experimental groups. MSC cells or C-MSC cells were trypsinized, and the cell concentration was adjusted to 4×10^4 cells/mL. Then, 100 μL of this cell suspension was inoculated into each well of a 96-well plate, each well containing an equal volume of hydrogel. The plates were incubated at standard conditions. At 24-, 48-, and 72-hours post-inoculation, 10 μL of CCK-8 reagent (Dojindo, Japan) was added to each well. After 2 hours of incubation, the absorbance at 450 nm was measured using a microplate spectrophotometer (BioTek, USA).

### Dead/Live staining

To examine the effects of hydrogels on cells, Live/Dead cell staining was performed on MSC cells from different experimental groups using the Live/Dead Cell Double Staining Kit (Dojindo, Japan) as per the manufacturer's instructions. MSC were incubated with HAMA, HAMA@MSC, and HAMA@C-MSC hydrogels, respectively, in an incubator for 48 hours. Following this, Calcein-AM and propidium iodide (PI) were added to the cultures, which were then incubated in the dark for 60 minutes. The cells were subsequently observed and photographed under a confocal microscope (Olympus, Japan).

### Hemolysis assay

HAMA and HAMA@C-MSC were incubated with a 4% rabbit erythrocyte suspension (Yuanye Bio-Technology, China) on a constant temperature shaker at 37°C for 4 hours. PBS was used as negative control, and water was used as the positive control. After incubation, the centrifuge tubes for each group were centrifuged at 3,500 rpm for 10 minutes. Digital photographs were taken to document hemolysis in the different groups. The supernatant was aspirated, and the absorbance at 570 nm was measured using a microplate reader (BioTek, USA) to calculate the hemolysis rate.

### Immunocytofluorescence

Cells were incubated with various drugs, after which 200 µL of culture medium and 200 µL of paraformaldehyde were added to each well for a 5-minute pre-fixation step. This was followed by the addition of 400 µL of paraformaldehyde per well to fully fix the cells for 10 minutes. After fixation, cells were washed three times with PBS. Cells were then permeabilized with PBS containing 0.3% Triton X-100 for 10 minutes at room temperature, followed by three additional washes with PBS. To block non-specific binding, cells were incubated with 300 µL of goat serum for 1 hour at room temperature. Cells were then incubated overnight at 4°C with primary antibodies: collagen type II alpha 1 chain (COL2A; 1:500, Cat# 28459-1-AP, Proteintech, USA), matrix metalloproteinase-13 (MMP13; 1:500, Cat# 18165-1-AP, Proteintech, USA), and caspase-3 (1:500, Cat# 9662, Cell Signaling Technology, USA). Following primary antibody incubation, cells were exposed to Alexa Fluor 488-conjugated secondary antibody (1:1000, Cat# ab150077, Abcam, USA) and Alexa Fluor 594-conjugated secondary antibody (1:1000, Cat# ab150080, Abcam, USA) for 2 hours at room temperature in the dark to detect the respective antigens. Nuclei were stained using Hoechst stain (Beyotime, China) to facilitate cell visualization. After a final series of three washes in PBS, the slides were mounted using an anti-fade mounting medium to preserve fluorescence. Samples were analyzed in a dark room using a laser confocal microscope (Olympus, Japan), and representative areas were photographed to assess cellular responses to the treatment.

### Quantitative real-time PCR (qRT-PCR)

For RNA isolation, 1 mL of lysis buffer (Takara, Japan) was added to each sample of cells or pulverized tissues, which were then mixed thoroughly using a vortexer. The samples were allowed to stand at room temperature for 5 minutes to ensure complete dissociation of nucleic acid-protein complexes. Chloroform (0.2 mL) was added to each sample, the tubes were capped, shaken vigorously for 15 seconds, and allowed to stand at room temperature for an additional 5 minutes. The samples were then centrifuged at 12,000 rpm for 10 minutes at 4°C in a pre-cooled centrifuge. The upper colorless aqueous phase containing RNA was carefully transferred to a new tube. RNA purification involved the addition of 500 μL of column wash solution to the adsorbent column, which was allowed to sit at room temperature for 2 minutes before being centrifuged at 12,000 rpm for 2 minutes at 4°C; the waste liquid was discarded. Anhydrous ethanol (200 μL) was added to the supernatant, which was transferred to the column, allowed to stand for 2 minutes, and then centrifuged at 12,000 rpm for 2 minutes at 4°C; the waste liquid was again discarded. Rinse solution (600 μL) was added to the column, which was centrifuged at 12,000 rpm for 2 minutes at 4°C, and the waste solution was discarded. This rinse step was repeated once more with an additional centrifugation for 5 minutes at 12,000 rpm at 4°C. The column was allowed to dry at room temperature for a few minutes to eliminate any residue of the rinse solution.

RNA was eluted by placing the column in a new tube, to which 100 μL of RNase-free ddH2O was added dropwise to the center of the membrane; the column was allowed to stand at room temperature for 5 minutes before being centrifuged at 12,000 rpm for 2 minutes at room temperature. cDNA was subsequently synthesized from the RNA according to the manufacturer's instructions (Takara, Japan).

The PCR reaction was prepared by adding 0.4 μL of forward primer, 0.4 μL of reverse primer, 10 μL of TB Green Premix Ex Taq II FAST qPCR (2×), 2 μL of cDNA, and 7.2 μL of ddH2O to PCR tubes. qRT-PCR was performed using an Applied Biosystems instrument (USA) with the cycling conditions comprising an initial denaturation at 95 °C for 30 seconds, followed by 40 cycles of 5 seconds at 95 °C and 30 seconds at 55 °C. The expression of target genes was quantified using the 2-ΔΔCT method, with GAPDH being used as the internal reference gene. Primer sequences are listed in Table S1.

### Western Blot

Total cellular proteins were extracted using cell lysate (Beyotime, China) containing protease inhibitors. Proteins were completely denatured by adding 5× Loading buffer (Beyotime, China) and boiling at 100°C for 10 minutes. Proteins were then separated by SDS-PAGE electrophoresis and transferred onto polyvinylidene difluoride (PVDF) membranes (Millipore, USA). The membranes were blocked using 5% skimmed milk powder to prevent non-specific binding. Overnight incubation at 4°C with diluted primary antibodies followed: ACAN (1:2000, Cat# 68350-1-Ig, Proteintech, USA), COL2A (1:2000, Cat# 28459-1-AP, Proteintech, USA), SOX9 (1:2000, Cat# 67439-1-Ig, Proteintech, USA), MMP13 (1:2000, Cat# 18165-1-AP, Proteintech, USA), CD9 (1:1000, Cat# 13174, CST, USA), CD63 (1:1000, Cat# 52090, CST, USA), GM130 (1:1000, Cat# 12480, CST, USA), Bcl-2 (1:1000, Cat# 3498, CST, USA), P53 (1:1000, Cat# 9282, CST, USA), and GAPDH (1:10000, Cat# 10494-1-AP, Proteintech, USA). The following day, the membranes were incubated with goat anti-rabbit IgG-HRP (1:10000, Cat# ab205718, Abcam, USA) and goat anti-mouse IgG-HRP (1:10000, Cat# ab205719, Abcam, USA) for 90 minutes. Bands were visualized and photographed using a chemiluminescence instrument (Bio-Rad, USA). Quantitative analysis of the bands was performed using ImageJ software.

### Cell apoptosis assay

For the flow cytometry-based apoptosis assay, 10× Binding Buffer was diluted with deionized water to achieve 1× Binding Buffer. After the collected cells were digested with EDTA-free trypsin, they were collected by centrifugation at 1000 rpm for 5 minutes at room temperature. The cells were then washed by resuspension in pre-cooled PBS (4°C) followed by centrifugation at 1000 rpm for 5 minutes. The cells were resuspended in 300 μL of 1× Binding Buffer. Subsequently, 5 μL of Annexin V-FITC (Dojindo, Japan) was added, mixed well, and incubated for 15 minutes at room temperature in the dark. 5 minutes before the end of the incubation, 5 μL of PI (Dojindo, Japan) staining solution was added. After incubation, an additional 200 μL of 1× Binding Buffer was added to each sample. Flow cytometry analysis was conducted using a flow cytometer (Beckman Coulter, USA), where Annexin V-FITC binding was analyzed using an FITC signal detector (FL1) and PI staining was detected by a propidium iodide signal detector (FL2). In the analysis, normal cells showed no staining (Annexin V-/PI-), cells in early apoptosis showed green fluorescence (Annexin V+/PI-), and late apoptotic or necrotic cells showed both green and red fluorescence (Annexin V+/PI+).

### Animals

SD rats aged 8-10 weeks were subjected to destabilization of the medial meniscus (DMM) surgery to construct in vivo osteoarthritis (OA) models. These experimental animals were acquired from Changzhou Cavins Laboratory Animal Co. During the surgery, the rats were anesthetized with isoflurane (RWD Life Science, China) using an inhalation anesthesia machine. The meniscus was carefully removed from the medial tibial plateau, after which various hydrogels were administered to the treatment groups. After the application of hydrogels, the surgical wounds were sutured. Two months post-surgery, all animals were euthanized, and their knee joints were harvested for further pathological analysis and tissue sectioning. The conduct of these animal experiments adhered strictly to the guidelines evaluated and approved by the Ethics Committee of Shanghai University.

### Micro-CT analysis

To visualize osteophytes in OA, knee joints from SD rats were harvested and fixed in paraformaldehyde (specify concentration, typically 4%) for 48 hours. Excess muscle tissue was carefully removed from the surrounding areas. The specified regions were then scanned using a micro-computed tomography system (Micro-CT, Bruker, Germany) at settings of 80 kV and 60 µA. Three-dimensional images of the rat knee joints were reconstructed using CTvol software.

### Arthropathologic analysis

After fixation, the knee joints were immersed in an EDTA-based tissue decalcification solution (specify concentration if critical) for 6 weeks to ensure complete decalcification before paraffin embedding. Tissue sections were then cut to the optimal thickness (specify thickness, e.g., 5 µm) for histological examination. All sections underwent standardized staining procedures according to the manufacturer's instructions. Hematoxylin and eosin (H&E) staining was used to assess general tissue structure and cellular details, while Safranin O/Fast Green (S&O) staining was applied to evaluate the damage and repair of articular cartilage.

### Immunohistochemistry and immunofluorescence analysis

For immunohistochemical staining of knee joint tissue sections, the sections were first deparaffinized and subjected to antigen retrieval using a sodium citrate solution (specify concentration, if critical) at 95°C for 15 minutes, followed by three washes with PBS. The sections were then permeabilized with 0.1% Triton X-100 in PBS for 15 minutes, followed by additional PBS washes. Blocking was performed using PBS containing 10% goat serum. The COL2A primary antibody (1:1000, Cat# 28459-1-AP, Proteintech, USA) was applied, and the sections were incubated overnight at 4°C. The next day, HRP-conjugated secondary antibody (1:1000, Cat# ab6721, Abcam, USA) was applied for 1 hour, followed by DAB substrate staining for 3 minutes. Images were captured using a bright-field microscope.

For immunofluorescent staining, sections were incubated overnight at 4°C with Caspase-3 primary antibody (1:1000, Cat# 9662, CST, USA). The following day, they were incubated with a 488 nm fluorescent secondary antibody (1:1000, Cat# ab150077, Abcam, USA) for 1 hour, followed by DAPI staining for 3 minutes to visualize nuclei. The sections were then mounted, and images were captured using a fluorescence microscope.

### Magnetic Resonance Imaging

The condition of rat knee joints was evaluated using a small animal magnetic resonance imaging (MRI) system (Bruker BioSpec 70/20 USR, Germany). This system is equipped with a 7T superconducting magnet, featuring a bore diameter of 20 cm and a maximum gradient strength of 446.1 mT/m. It achieves a spatial resolution limit of 0.065 × 0.065 × 0.065 mm. The arthritic knee joints of the rats were immobilized within the system for imaging. Imaging parameters were carefully set for both T2- and T1-weighted sequences to optimize visualization of the joint structure. For the T2-weighted sequence, the parameters included an echo time (TE) of 30 ms, repetition time (TR) of 3000 ms, slice thickness of 0.6 mm, and a field of view (FOV) of 30 × 29.56 mm. The T1-weighted sequence parameters were set with a TE of 4.0 ms, TR of 300 ms, and the same slice thickness and FOV as the T2 sequence.

### ELISA

For cell culture supernatant samples, the samples were collected and centrifuged at 4°C and 1000 × g for 20 minutes to remove impurities and cell debris. For tissue samples, the tissues were rinsed with pre-cooled PBS (0.01M, pH=7.4) to remove any residual blood, weighed, and then minced. The minced tissues were then homogenized in a metal homogenizer with PBS containing protease inhibitors at a 1:9 ratio and ground thoroughly on ice. The homogenate was subsequently centrifuged at 4°C and 5000 × g for 10 minutes, and the supernatant was collected for analysis. To proceed with the analysis, 100 μL of a doubly diluted standard was added to the standard wells, 100 μL of sample dilution was added to the blank wells, and 100 μL of the sample to be tested was added to the remaining wells. The wells were sealed with a plate-sealing film and incubated at 37°C for 90 minutes. After incubation, the liquid was removed from the wells, and 100 μL of Biotinylated Antibody Working Solution was added to each well. The plate was then sealed and incubated again at 37°C for 1 hour. Following this, the plate was washed three times with 300 μL of washing solution after carefully patting the wells dry with clean absorbent paper. Next, 100 μL of HRP enzyme conjugate solution was added to each well, and the plate was incubated at 37°C for 30 minutes, then washed five times as previously described. Subsequently, 90 μL of TMB solution was added to all wells, and the plate was sealed with a membrane and incubated at 37°C for 15 minutes. Finally, 50 μL of stop solution was added, and the absorbance of each well was measured immediately at 450 nm using a microplate reader (BioTek, USA). The concentration of the target protein in each sample group was calculated against the standard curve.

### Statistical analysis

Each experimental value was expressed as the mean ± standard deviation. The data normality was determined using a Shapiro-Wilks test. For comparisons between two groups, an unpaired two-tailed t-test was performed. One-way analysis of variance (ANOVA) was utilized for pairwise comparisons among multiple groups, with post hoc tests conducted to adjust for multiple comparisons as necessary. Significance levels were set at *p < 0.05, **p < 0.01, and ***p < 0.001. Values where p > 0.05 were indicated as no significant difference (ns). All statistical analyses were conducted using Excel 2016 and GraphPad Prism 9.

**Figure 2.**
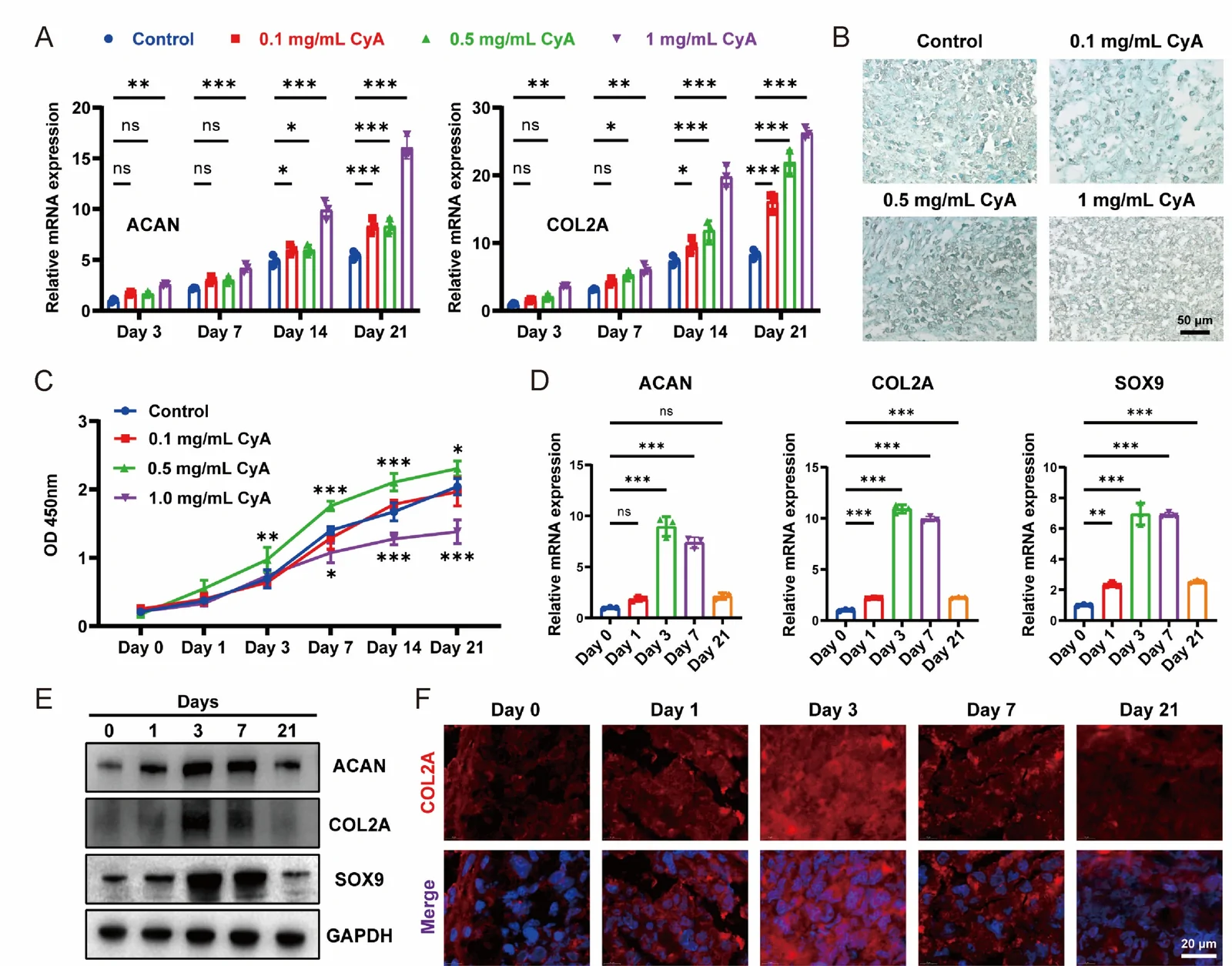
**CyA promotes chondrogenic differentiation of MSC**. (**A**) Relative mRNA expression levels of ACAN and COL2A in MSC. (**B**) Alcian blue staining images of MSC cultured with CyA for 21 days. (**C**) Cell viability of MSC over 21 days. (**D**) Relative mRNA expression levels of ACAN, COL2A, and SOX9 in MSC over 21 days. (**E**) Protein expression levels of ACAN, COL2A, and SOX9 in MSC over 21 days. (**F**) Representative immunocytochemistry images of COL2A in MSC from Day 0 to Day 21. Statistical significance was determined using one-way ANOVA for multiple group comparisons, followed by post hoc tests. All data are presented as mean ± SD (n = 3). *p < 0.05, **p < 0.01, ***p < 0.001, and ns = no statistically significant difference between groups.

**Figure 3.**
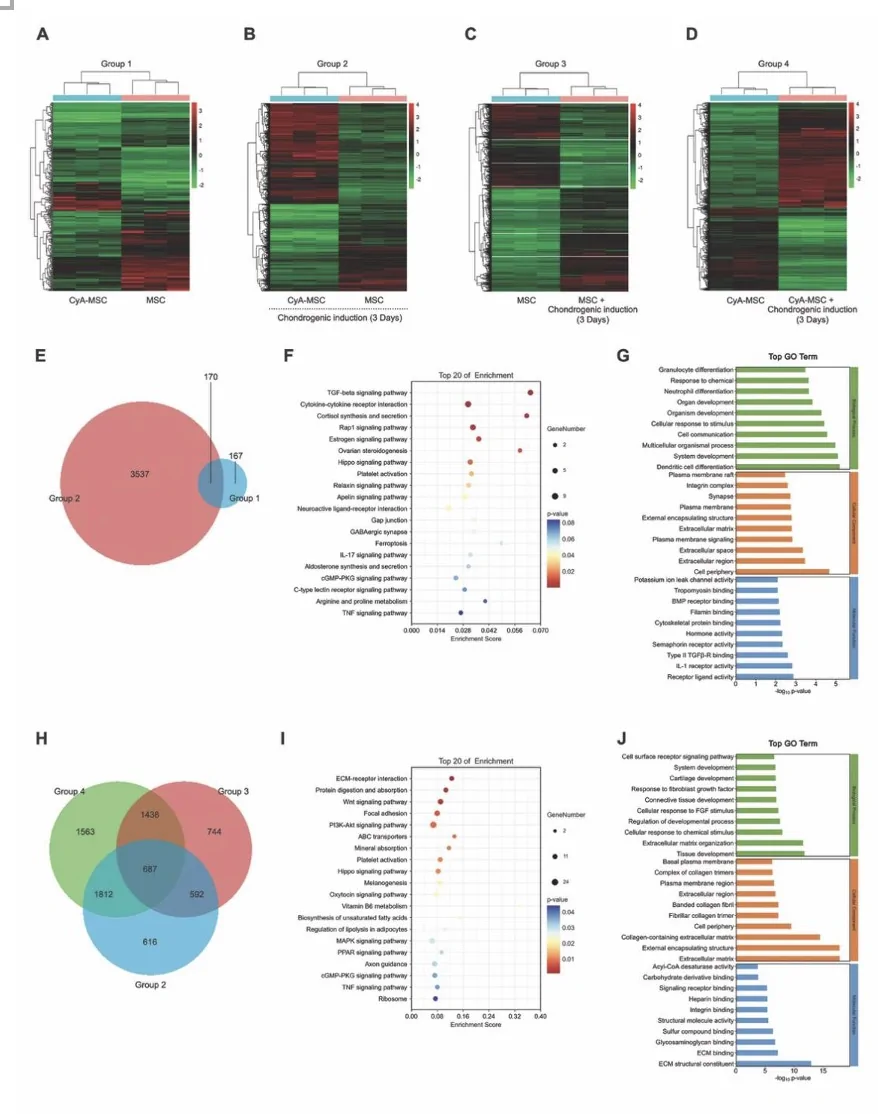
**Transcriptome sequencing of chondrogenic differentiation in C-MSC and MSC**. (**A**) Heatmap of differentially expressed genes between C-MSC and MSC. (**B**) Heatmap of differentially expressed genes between C-MSC and MSC after 3 days of chondrogenic induction. (**C**) Heatmap of differentially expressed genes between MSC and MSC after 3 days of chondrogenic induction. (**D**) Heatmap of differentially expressed genes between C-MSC and C-MSC after 3 days of chondrogenic induction. (**E**) Venn diagram showing common differentially expressed genes between Group 1 and Group 2. (**F**) KEGG enrichment analysis of 170 common differentially expressed genes between Group 1 and Group 2. (**G**) GO enrichment analysis of 170 common differentially expressed genes between Group 1 and Group 2. (**H**) Venn diagram showing common differentially expressed genes among Group 2, Group 3, and Group 4. (**I**) KEGG enrichment analysis of 687 common differentially expressed genes among the three groups. (**J**) GO enrichment analysis of 687 common differentially expressed genes among the three groups.

## RESULTS

### Screening Study on Drug Concentration and Time Gradient of CyA-induced MSC

The induction of mesenchymal stem cells (MSC) with Cyaonoside A (CyA) was investigated at varying concentrations and time points to assess its effects on chondrogenic differentiation. The real-time quantitative PCR (RT-qPCR) analysis revealed a dose-dependent increase in the expression of the key chondrogenic genes, ACAN and COL2A. Notably, the gene expression dramatically increased across the timeline, with the most significant enhancements observed during the initial stages of chondrogenic differentiation (Fig. 2A).

Histological assessments conducted on cartilage balls formed after 21 days demonstrated optimal cell structure and morphology at a CyA concentration of 0.5 mg/mL. These sections showed tightly arranged cells with denser nuclei and overall, more perfected morphology and function compared to other concentrations. In contrast, at 1 mg/mL CyA, despite higher gene expression levels, the morphological assessment through electron microscopy did not reveal improved outcomes, suggesting a detrimental effect of high CyA doses on cell differentiation and viability (Fig. 2B and Supplementary Fig. 1).

Cytotoxicity assays using CCK-8 indicated that 0.5 mg/mL CyA supported cell viability and even enhanced MSC proliferation, while 1 mg/mL CyA exhibited a reductive effect on cell growth over the same period (Fig. 2C). These findings underscore the importance of optimizing CyA concentration to balance gene expression enhancements with potential cytotoxic effects.

Further analysis focused on determining the optimal time point for CyA-induced differentiation. The analysis conducted over 21 days showed that the expression of chondrogenic genes SOX9, ACAN, and COL2A peaked significantly on Day 3. This peak was statistically significant when compared to control and other time points, as demonstrated by both qPT-PCR and Western blot analysis (Fig. 2D and 2E). Additionally, COL2A fluorescence staining of the cartilage slices confirmed the most pronounced staining and chondrogenic activity on Day 3, indicating robust differentiation by this point (Fig. 2F).

The comprehensive evaluation established 0.5 mg/mL of CyA and a 3-day induction period as the optimal conditions for promoting chondrogenic differentiation in MSC. These optimized parameters have been identified as the most effective for advancing cartilage regeneration studies and will be used as a standard for subsequent experiments.

### Analysis of the Pharmacological Effects and Chondrogenic Differentiation Mechanism of CyA-induced MSC

The analysis began by comparing C-MSC with unmodified MSC (Group 1), revealing 337 differential genes, which indicated an immediate impact of CyA on gene expression (Fig. 3A). To further investigate the effects of CyA, gene expression profiles were compared across three groups: C-MSC vs. MSC after 3 days of chondrogenic induction (Group 2), MSC vs. MSC after 3 days of chondrogenic induction (Group 3), and C-MSC vs. C-MSC after 3 days of chondrogenic induction (Group 4). The analysis revealed 3,707 differential genes in Group 2, 3,461 in Group 3, and 5,500 in Group 4, underscoring the crucial role of CyA in regulating gene expression during the process of chondrogenic differentiation (Fig. 3B-D).

Subsequent analysis using a Venn diagram identified 170 genes common between the initial and Day 3 differential gene profiles of C-MSC and MSC. These genes are considered crucial for the chondrogenic effects of CyA (Fig. 3E). Pathway analysis via Kyoto Encyclopedia of Genes and Genomes (KEGG) of the top 20 significantly enhanced genes revealed their involvement in critical pathways such as TGF-β signaling, cytokine-cytokine receptor interaction, and estrogen signaling, all essential for chondrogenic processes (Fig. 3F). Additionally, Gene Ontology (GO) analysis showed that CyA impacts organ development, particularly affecting structures like integrin complexes and the extracellular matrix (Fig. 3G).

An extended analysis revealed 687 differential gene interactions, offering deeper insights into the role of CyA in chondrogenic differentiation. These interactions were primarily involved in pathways like PI3K-Akt, which are essential for cell growth and survival (Fig. 3H). Continuing the comparative analysis over three days within a chondrogenic environment (Group 2) confirmed CyA's profound influence on gene expression, notably altering pathways related to osteoarthritis (OA) treatment such as ECM receptor interaction and Wnt signaling (Fig. 3I). This extensive gene interaction study also indicated significant changes in biological processes crucial for cartilage development and connective tissue structuring (Fig. 3J).

The comprehensive analysis confirms that CyA dramatically enhances the chondrogenic potential of MSC, with optimal effects observed at a concentration of 0.5 mg/mL and an induction period of 3 days. These findings not only provide a molecular basis for understanding CyA's efficacy but also highlight its therapeutic potential for enhancing cartilage regeneration in OA treatments. Future studies are encouraged to explore the clinical applicability of these findings in human models.

**Figure 4.**
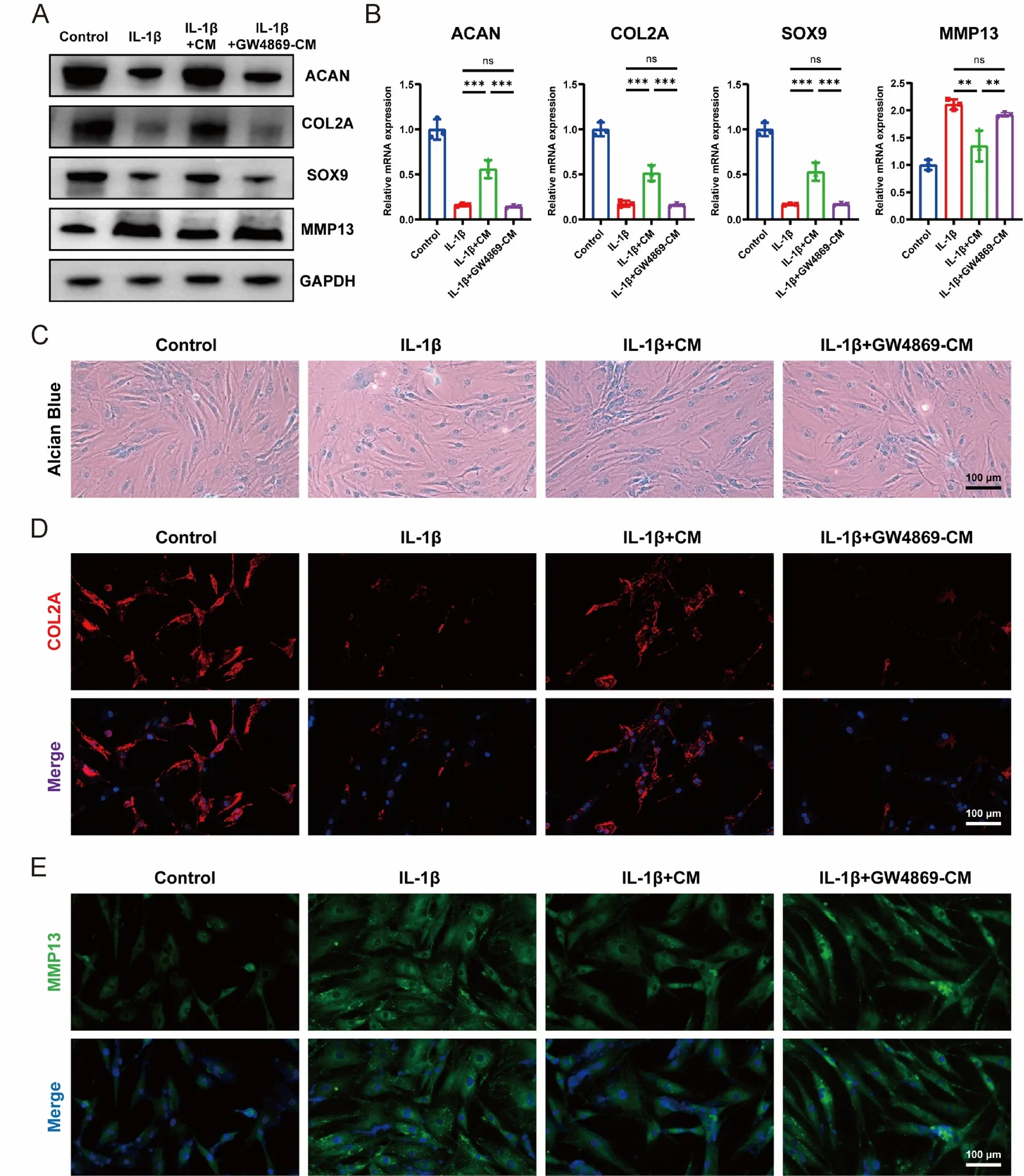
**Expression of ACAN, COL2A, SOX9, and MMP13 in chondrocytes**. (**A**) Western blot analysis of ACAN, COL2A, SOX9, and MMP13 expression in chondrocytes. (**B**) Relative mRNA expression levels of ACAN, COL2A, SOX9, and MMP13 in chondrocytes. Statistical significance was determined using one-way ANOVA for multiple group comparisons, followed by post hoc tests. All data are presented as mean ± SD (n = 3). **P < 0.01, ***P < 0.001, and ns = no statistically significant difference between groups. (**C**) Alcian blue staining images of chondrocytes. (**D**) Immunofluorescence images of COL2A in chondrocytes. (**E**) Immunofluorescence images of MMP13 in chondrocytes.

**Figure 5.**
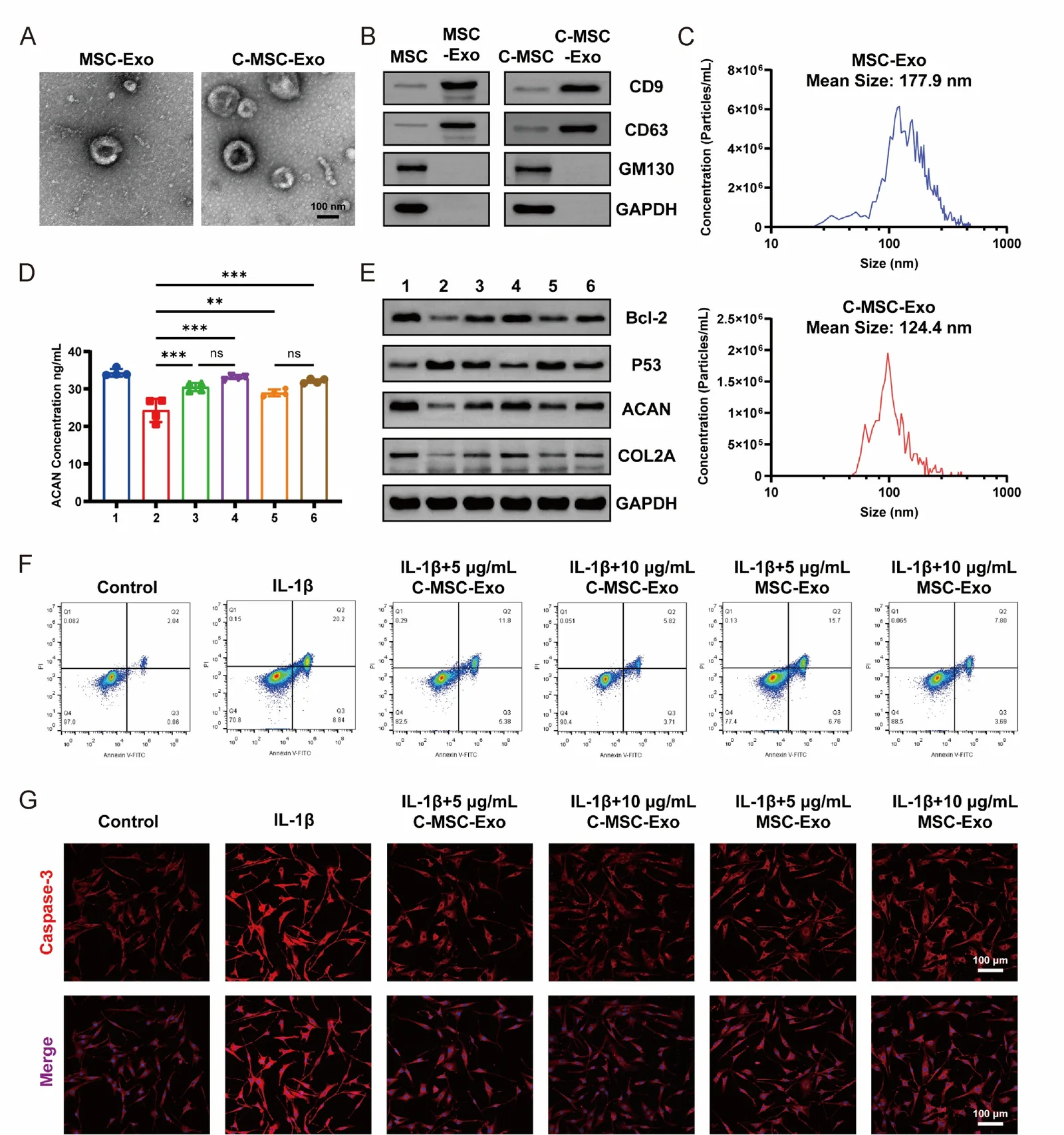
**Extraction and characterization of C-MSC exosomes**. (**A**) Representative TEM image of exosomes. (**B**) Western blot analysis of exosome markers. (**C**) Particle size analysis of exosomes. (**D**) Recovery of cartilage synthesis gene expression by C-MSC exosomes. (**E**) Recovery of cartilage synthesis protein expression by C-MSC exosomes. (**F**) C-MSC exosomes inhibit chondrocyte apoptosis. (**G**) Detection of Caspase3 expression in human chondrocytes using LSCM across different groups. Statistical significance was determined using one-way ANOVA for multiple group comparisons, followed by post hoc tests. All data are presented as mean ± SD (n = 4). **P < 0.01, ***P < 0.001, and ns = no statistically significant difference between groups.

### CyA-induced MSC Restores the Function of Chondrocytes with Reduced Inflammation

Human chondrocytes were prepared from patients undergoing total knee replacement and subsequently co-cultured with C-MSC supernatant to evaluate the effects on apoptosis induced by interleukin-1 beta (IL-1β). The experimental setup included four groups: a control group of chondrocytes in normal medium, a group exposed to IL-1β, a group co-cultured with C-MSC supernatant and IL-1β (IL-1β+CM), and a group treated with both an exosome inhibitor (GW4869) and the C-MSC supernatant alongside IL-1β (IL-1β+GW4869+CM).

Western blot analysis revealed a significant decrease in the expression of chondrogenic genes ACAN, COL2A, and SOX9 in the IL-1β group, along with an increase in the cartilage degradation gene MMP13. In contrast, the IL-1β+CM group showed a significant recovery in the levels of ACAN, COL2A, and SOX9, and a reduction in MMP13 expression, suggesting a protective effect of the C-MSC supernatant on cartilage. Conversely, the IL-1β+GW4869+CM group exhibited gene expression patterns similar to the IL-1β group, indicating that the protective effects of the C-MSC supernatant were mitigated by the inhibition of exosomes (Fig. 4A and Supplementary Fig. 3).

RT-qPCR results corroborated the Western blot findings, showing upregulation of ACAN, COL2A, and SOX9 in the IL-1β+CM group compared to the groups treated with the exosome inhibitor, alongside inhibition of MMP13 (Fig. 4B). Alcian blue staining demonstrated that chondrocytes in the IL-1β+CM group were denser and exhibited more complete morphology than those in the IL-1β or IL-1β+GW4869+CM groups (Fig. 4C). Fluorescence staining for COL2A revealed significant recovery in its expression in the IL-1β+CM group compared to the marked decrease observed in the IL-1β and IL-1β+GW4869+CM groups (Fig. 4D). Additionally, MMP13 fluorescence staining showed a notable decrease in the IL-1β+CM group, further supporting the protective role of C-MSC supernatant exosomes (Fig. 4E).

The findings from this study clearly demonstrate that C-MSC supernatant significantly mitigates IL-1β-induced apoptosis in human chondrocytes. The protective effects are primarily mediated by exosomes within the supernatant, which enhance the expression of chondrogenic genes and inhibit cartilage degradation pathways. These results suggest that C-MSC supernatant, particularly its exosome components, holds considerable therapeutic potential for improving cartilage regeneration and treating inflammatory conditions in OA.

### CyA-induced MSC Exosomes Improve Chondrocyte Function and Reduce Inflammation

The morphological characterization of exosomes from both MSC and C-MSC showed that they maintained spherical structures, as observed under a transmission electron microscope (Fig. 5A). Western blot analysis confirmed the presence of exosome-specific proteins CD9 and CD63 in both exosome types (Fig. 5B). Particle size analysis indicated that C-MSC exosomes had a smaller average particle size (124.6 nm) compared to MSC exosomes (177.9 nm), with a more concentrated size distribution, suggesting higher tissue permeability and potential for enhanced tissue repair (Fig. 5C).

Exosomes were added to human chondrocytes treated with IL-1β for 24 hours, and the cultures were continued for an additional 48 hours. The groups included control (normal medium), IL-1β alone, and cells treated with varying concentrations of C-MSC or MSC exosomes (5 μg/mL and 10 μg/mL). RT-qPCR analysis revealed that all exosome-treated groups showed restoration in the expression of key chondrogenic genes ACAN and COL2A, with a dose-dependent increase in expression levels (Supplementary Fig. 4). The group treated with 10 μg/mL C-MSC exosomes demonstrated the most significant improvement and was statistically different from the equivalent MSC exosome group, indicating a superior effect of C-MSC exosomes in reducing inflammatory damage (Fig. 5D and Supplementary Fig. 5).

Western blot results further supported the gene expression findings, showing increased levels of chondrogenic proteins ACAN and COL2A proportional to the concentration of C-MSC exosomes added (Fig. 5E and Supplementary Fig. 6). The anti-apoptosis protein Bcl-2 was found to increase, and the cartilage degradation protein P53 decreased in exosome-treated groups, with the strongest effects observed in the 10 μg/mL C-MSC exosome group (Fig. 5E).

Flow cytometry for apoptosis detection revealed that chondrocytes in the IL-1β group exhibited significant apoptosis, which was substantially reduced in the groups treated with C-MSC exosomes, especially at the higher concentration of 10 μg/mL (Fig. 5F and Supplementary Fig. 7). Laser Scanning Confocal Microscopy showed a similar pattern for the apoptosis gene Caspase3, with its expression significantly reduced in the C-MSC exosome groups, indicating a potent anti-apoptotic effect of C-MSC exosomes compared to MSC exosomes (Fig. 5G).

These results demonstrate that C-MSC exosomes effectively inhibit apoptosis and protect against cartilage degradation in an inflammatory environment. The beneficial effects of C-MSC exosomes on chondrocytes are dose-dependent and surpass those of standard MSC exosomes, highlighting their potential as a therapeutic agent for treating inflammatory damage in cartilage. This suggests that C-MSC exosomes could be a valuable tool in regenerative medicine, particularly in the context of OA treatment.

### Construction and Characterization of Hydrogel Loaded with CyA-Induced MSC

**Figure 6.**
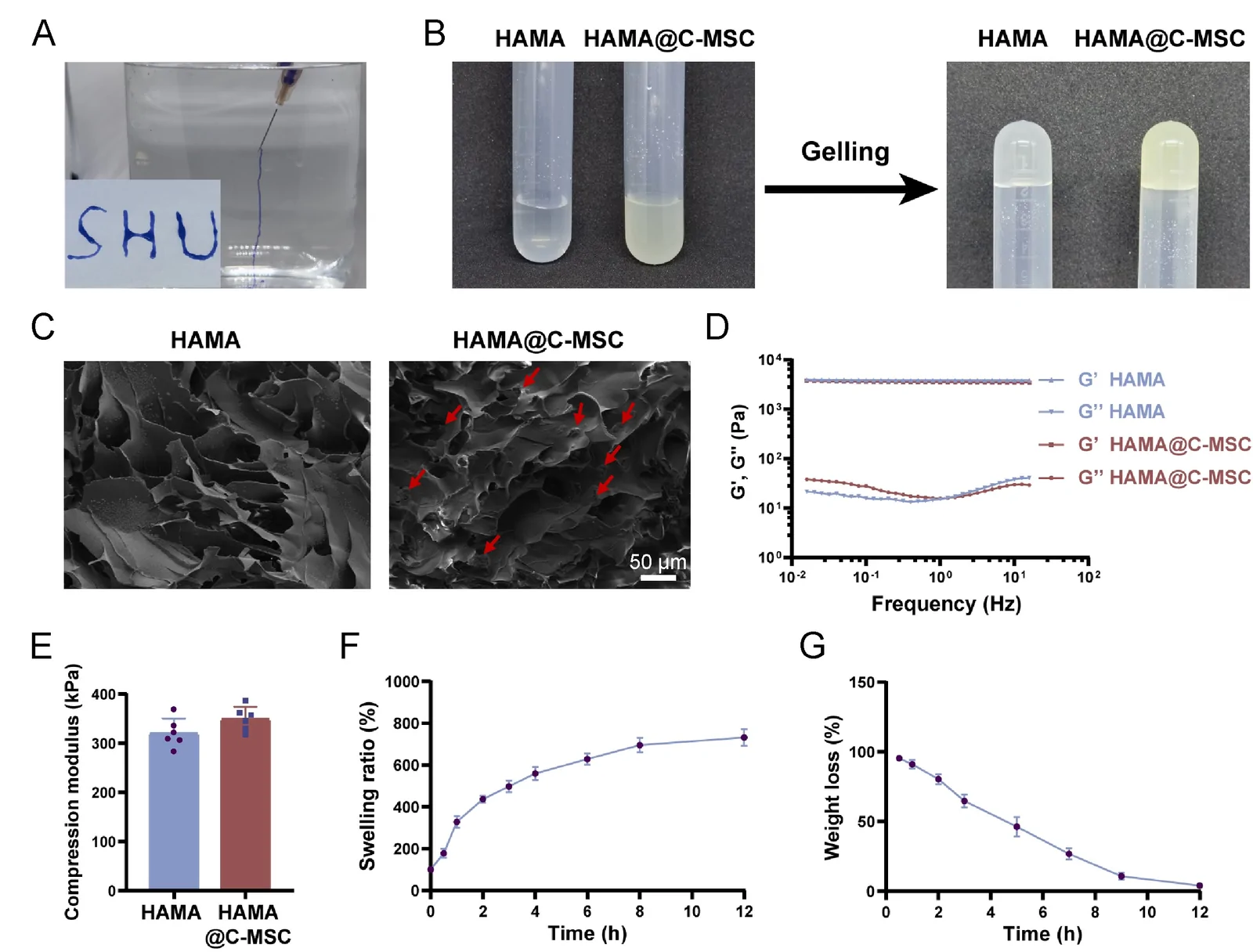
**Construction and characterization of HAMA@C-MSC hydrogel**. (**A**) Injectability of HAMA@C-MSC hydrogel. (**B**) Bright-field image of HAMA@C-MSC hydrogel. (**C**) Representative SEM image of HAMA@C-MSC hydrogel. (**D**) Rheological properties of HAMA@C-MSC hydrogel. (**E**) Compressive modulus of HAMA@C-MSC hydrogel. Data are presented as mean ± SD (n = 6). (**F**) Swelling ratio of HAMA@C-MSC hydrogel. (**G**) Degradation rate of HAMA@C-MSC hydrogel.

An injectable hydrogel system using methylacrylated hyaluronic acid (HAMA) was developed for targeted joint therapy with C-MSC. Hyaluronic acid (HA), a key extracellular matrix component, is abundant in synovial fluid and supports joint function through its viscoelasticity. To address HA’s rapid degradation, methacrylic anhydride was introduced to synthesize HAMA, enhancing its stability, mechanical properties, and suitability for joint repair. The HAMA hydrogel was prepared in a photosensitive formulation that solidifies under ultraviolet irradiation, transitioning from a translucent flowable state to an opaque non-flowable gel (Fig. 6A-B). The structural integrity and distribution of C-MSC within the hydrogel were confirmed through scanning electron microscopy, which showed that cells were evenly distributed along the pore walls of the scaffold without altering the hydrogel's structure (Fig. 6C).

Rheological analysis demonstrated that the incorporation of C-MSC into the hydrogel did not significantly affect its mechanical properties, as indicated by the similar compression modulus of HAMA and HAMA@C-MSC (Fig. 6D-E). Additionally, swelling and degradation performance tests indicated that the HAMA@C-MSC hydrogel exhibited good swelling capacity and degradation rates, suitable for sustained therapeutic applications (Fig. 6F-G).

This comprehensive approach to developing a cell delivery system using HAMA highlights its potential in enhancing the reparative capabilities of C-MSC in joint treatment, combining the inherent biological functionalities of HA with the regenerative properties of MSC. The findings suggest that HAMA-based hydrogels can serve as an effective platform for localized cell therapy in orthopedic applications, particularly in the management of joint-related conditions.

### Biocompatibility and Biosafety Evaluation of HAMA@C-MSC

**Figure 7.**
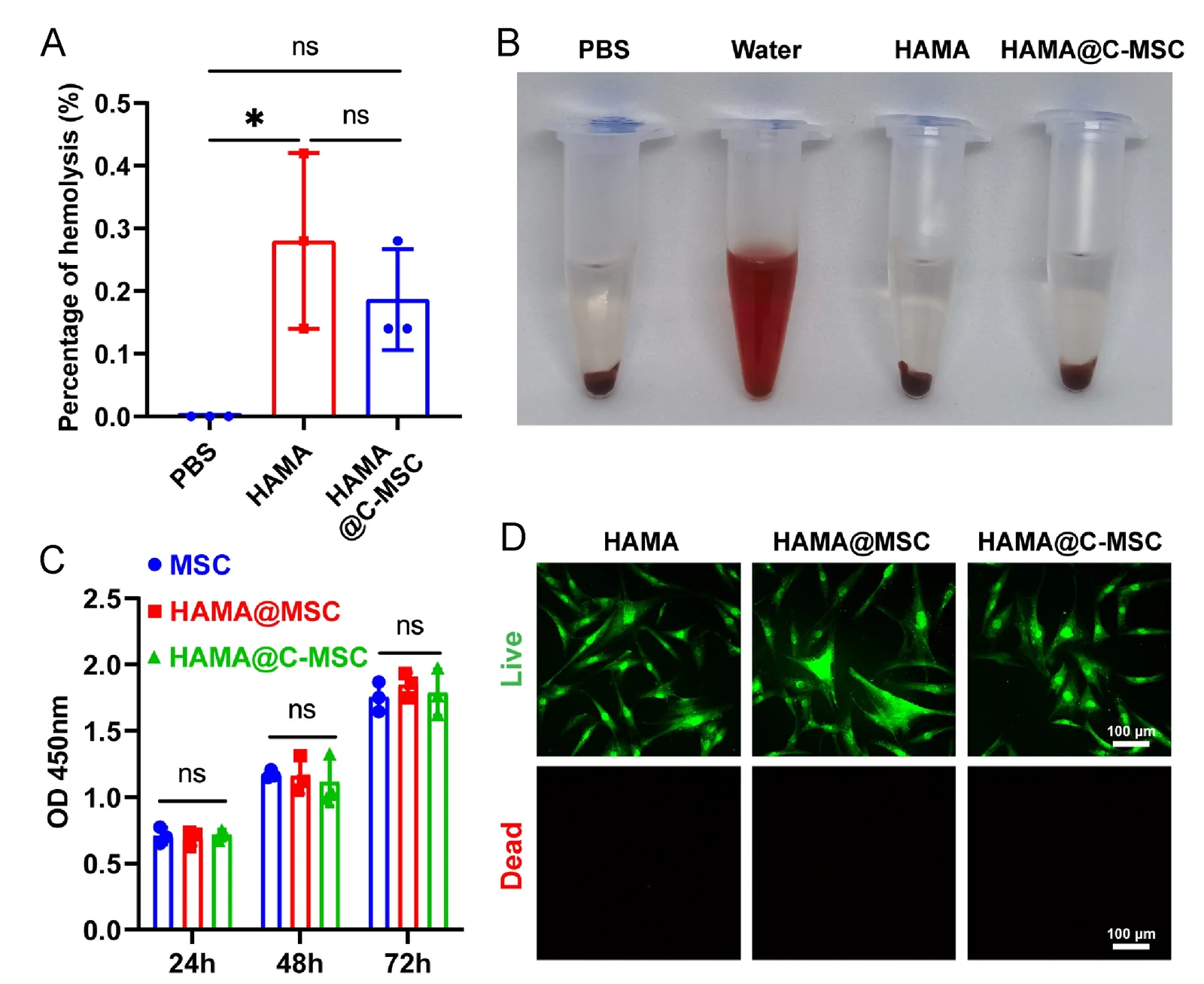
**Biosafety evaluation of HAMA@C-MSC hydrogel**. (**A**) Quantitative analysis of hemolysis. (**B**) Bright-field image of hemolysis detection. (**C**) Cytotoxicity assessment of HAMA@C-MSC hydrogel on MSC. (**D**) Live/dead staining of MSC treated with HAMA@C-MSC hydrogel. Statistical significance was determined using one-way ANOVA for multiple group comparisons, followed by post hoc tests. All data are presented as mean ± SD (n = 3). *p < 0.05, and ns = no statistically significant difference between groups.

To assess the biocompatibility of the HAMA@C-MSC delivery system, particularly regarding its direct interaction with articular cartilage upon injection into the joint cavity, several experiments were conducted. A hemolysis test was performed to evaluate the potential cytotoxicity of the hydrogel on red blood cells. The results indicated that both the blank HAMA hydrogel and the HAMA@C-MSC hydrogel did not cause significant rupture of rat red blood cells, suggesting that the materials do not induce hemolysis (Fig. 7A-B).

Further, the proliferation of MSC embedded within the hydrogels was assessed using a CCK-8 assay at multiple time points (24, 48, and 72 hours). The growth trends of MSC cultured in the HAMA hydrogel were compared with those of C-MSC in standard culture conditions. No significant differences were observed among the MSC group, the HAMA@MSC group, and the HAMA@C-MSC group, indicating that the hydrogel does not adversely affect MSC proliferation (Fig. 7C).

Additionally, a live/dead staining assay was performed to directly visualize cell viability within the hydrogels. This staining protocol uses green fluorescence to mark live cells and red fluorescence for dead cells. The results demonstrated that there were no observable differences in the viability of chondrocytes within the three different hydrogel formulations (HAMA, HAMA@MSC, and HAMA@C-MSC) after 48 hours, confirming the non-toxic nature of these materials (Fig. 7D).

Based on these experiments, the HAMA@C-MSC delivery system has been validated to possess excellent biosafety profiles, underlining its potential for safe application in joint therapy. The results affirm that this hydrogel-based delivery system is highly biocompatible, supporting its use for sustained delivery of therapeutic cells in treating joint disorders.

**Figure 8.**
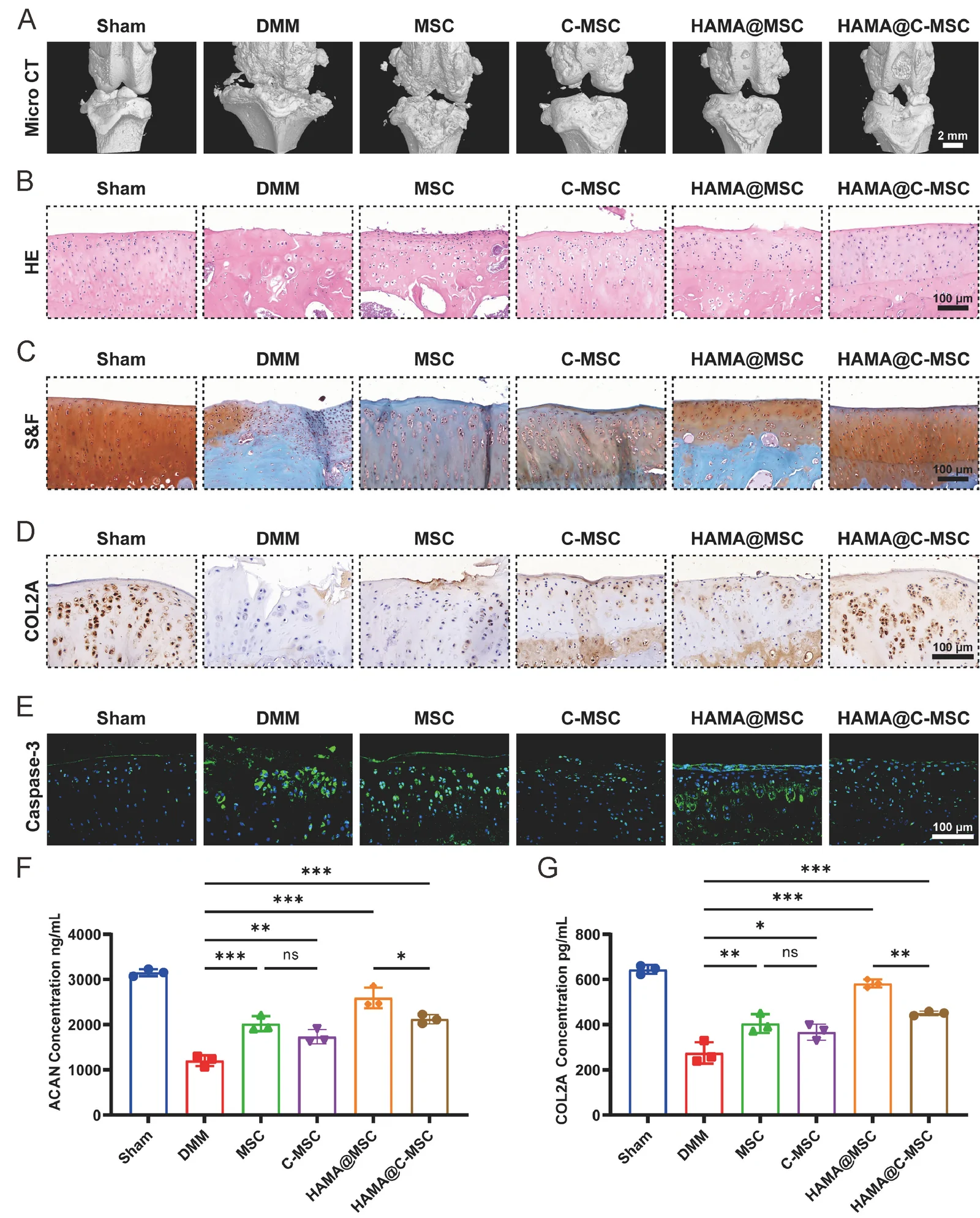
**In vivo therapeutic evaluation of hydrogel loaded with CyA-induced MSC**. (**A**) Representative 3D reconstruction images of joints in different groups. (**B**) H&E staining of articular cartilage in different groups. (**C**) S&F staining of articular cartilage in different groups. (**D**) Immunohistochemical staining of COL2A in articular cartilage across different groups. (**E**) Immunofluorescence staining of Caspase-3 in articular cartilage across different groups. (F, G) Relative mRNA expression levels of ACAN and COL2A in joint tissues of different groups. Statistical significance was determined using one-way ANOVA for multiple group comparisons, followed by post hoc tests. All data are presented as mean ± SD (n = 3). *p < 0.05, **p < 0.01, ***p < 0.001, and ns = no statistically significant difference between groups.

### *In Vivo* Verification of Therapeutic Efficacy of HAMA@C-MSC

To verify the therapeutic efficacy of the HAMA@C-MSC delivery system in vivo, male Sprague-Dawley (SD) rats aged 8 weeks and weighing between 220-250g were utilized. The rats were divided into six groups: control, model (DMM model of knee OA), MSC, C-MSC, HAMA@MSC, and HAMA@C-MSC. Four weeks after the induction of the DMM model, the respective treatments were injected into the rat knee joints once a week for four weeks with a dosage of 10^6^ cells/20µl. The HAMA@MSC and HAMA@C-MSC groups received additional ultraviolet irradiation for 30 seconds post-injection to solidify the hydrogel.

Four weeks post-treatment, the knee joints were harvested for analysis. Micro CT imaging indicated that the HAMA@C-MSC group maintained a more complete and normal joint structure, with smoother arcs on the tibial plateau and femoral trochlear surfaces compared to the other groups, which showed rough surfaces with abnormal osteophyte hyperplasia and evident wear (Fig. 8A). MRI scans further supported these findings, demonstrating that the HAMA@C-MSC group exhibited more uniform knee joint spaces and intact cartilage tissue, with biomechanical alignment closer to normal compared to other treatment groups (Supplementary Fig. 8).

Histological and immunological assessments were conducted to evaluate tissue regeneration and inflammation response. Hematoxylin and eosin (H&E) staining revealed that in the HAMA@C-MSC group, the surface of the tibial plateau cartilage appeared smooth and intact, with clearly differentiated layers of hyaline cartilage, fibrocartilage, and calcified cartilage (Figure 8B). Safranin O/Fast green (S&F) staining showed that the chondrocytes in the HAMA@C-MSC group were evenly arranged longitudinally, with a rich collagen matrix and smooth cartilage surface. In contrast, while the MSC, C-MSC, and HAMA@MSC groups showed some restoration in cartilage arrangement, the collagen matrix was not effectively restored (Fig. 8C).

Immunohistochemistry demonstrated a high expression of COL2A in the HAMA@C-MSC group, indicating robust cartilage repair and integrity of the cartilage surface layer (Fig. 8D). Additionally, fluorescence staining for Caspase-3 showed that the expression of apoptotic proteins was significantly lower in the HAMA@C-MSC group, suggesting an enhanced ability to inhibit cartilage apoptosis (Fig. 8E). RT-qPCR results confirmed the superior expression levels of cartilage-specific genes ACAN and COL2A in the HAMA@C-MSC group (Fig. 8F-G and Supplementary Fig. 9).

These comprehensive in vivo findings underscore that the HAMA@C-MSC delivery system not only improves cartilage surface smoothness and chondrocyte regeneration but also effectively restores collagen integrity in the joint, surpassing the effects observed in other treatment groups. This confirms that HAMA@C-MSC holds significant therapeutic potential for the treatment of OA, offering substantial improvements over traditional MSC-based therapies.

## DISCUSSION

This study establishes that the optimal induction regimen for CyA-induced MSC (C-MSC) involves using a concentration of 0.5 mg/mL for three days. The mRNA analysis reveals that C-MSC have a superior pharmacological effect over traditional MSC, particularly in activating pathways such as TGF-β signaling, cortisol synthesis and secretion, and Rap1 signaling. In the chondrogenic process, C-MSC outperform traditional MSC, especially in the Wnt and PI3K-Akt signaling pathways, demonstrating stronger gene expression related to cartilage and connective tissue development.

Furthermore, C-MSC excel in promoting fibrous collagen formation and glycosaminoglycan binding, indicating significant advantages in osteoarthritis (OA) treatment. The enhanced performance of C-MSC in various signaling pathways and molecular mechanisms underscores their potential over traditional MSC in chondrogenesis.

The study also investigates the therapeutic effects of C-MSC on OA, focusing on their ability to restore and protect chondrocyte function. Experiments confirm that C-MSC, particularly through the release of exosomes, effectively up-regulate chondrogenic genes and down-regulate apoptotic genes. This reversal of chondrocyte apoptosis and promotion of cartilage repair highlight the significant protective effects of C-MSC in inflammatory conditions.

By encapsulating C-MSC in HAMA hydrogel and administering them via in situ injection into the joint cavity, the study demonstrates their substantial therapeutic benefits in treating OA. Compared to traditional MSC, C-MSC shows enhanced cartilage protection and repair capabilities. Despite traditional Chinese medicine's historical contributions to clinical practice, its mechanisms often remain less understood in Western medicine [24]. This study not only showcases the potential of C-MSC, a new type of stem cell induced by a traditional Chinese medicine monomer, but also provides a theoretical basis for using such cells in OA treatment, paving the way for further research and application [25].

## Supplementary Materials

The Supplementary data can be found online at: www.aginganddisease.org/EN/10.14336/AD.2024.10016.
