# Supplementary material for: Enhanced Chondrogenic Potential and Osteoarthritis Treatment Using Cyaonoside A-Induced MSC Delivered via a Hyaluronic Acid-Based Hydrogel System
Source: Aging Dis. 2025 Jan 29;17(1):466–82. doi: 10.14336/AD.2024.10016 (PMC12727091; doi:10.14336/AD.2024.10016)
Supplement: Supplementary file 1 — The Supplementary data can be found online at: www.aginganddisease.org/EN/10.14336/AD.2024.10016. [file AD-17-1-466-s.pdf]

SUPPLEMENTARY DATA

Enhanced Chondrogenic Potential and Osteoarthritis Treatment Using Cyaonoside A-Induced MSC Delivered *via* a Hyaluronic Acid-Based Hydrogel System

Xingyan An, Qirong Zhou, Shihao Sheng, Anfu Deng, Han Liu, Xiuhui Wang, Qin Zhang, Yingying Jing, Ke Xu, Chongru He, Robert Chunhua Zhao, Jiacan Su

© 2025. An X et al. Published online at http://www.aginganddisease.org/EN/10.14336/AD.2024.10016

SUPPLEMENTARY DATA

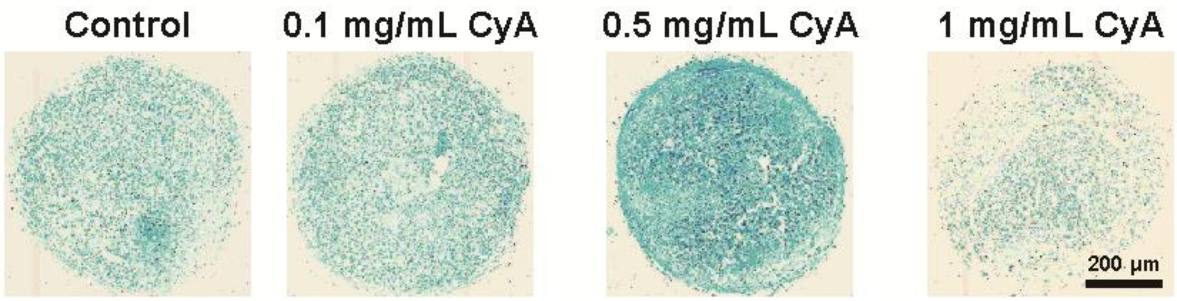

Supplementary Figure 1. Alcian blue staining of MSCs cultured with CyA for 21 days, as depicted in the overall view in Figure 2B.

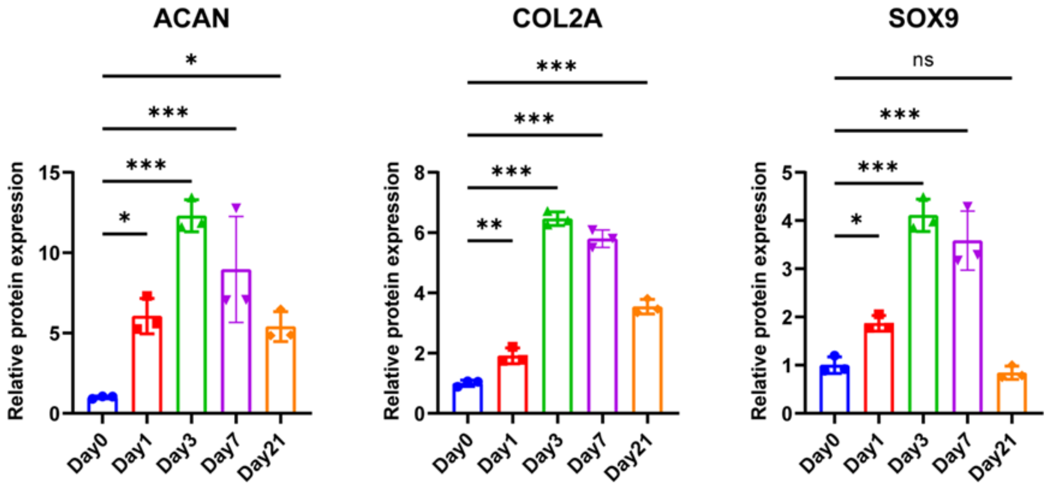

Supplementary Figure 2. Protein expression levels after CyA treatment. Western blot quantitative analysis shows that 0.5 mg/mL CyA induces the highest expression levels of the chondrogenic-related proteins SOX9, ACAN, and COL2A in MSCs on Day 3, compared to other time points. Statistical significance was assessed using one-way ANOVA for multiple group comparisons, followed by post hoc tests. All data are presented as mean ± SD (n=3). *p < 0.05, **p < 0.01, *p < 0.001, and ns = no statistically significant difference between groups.

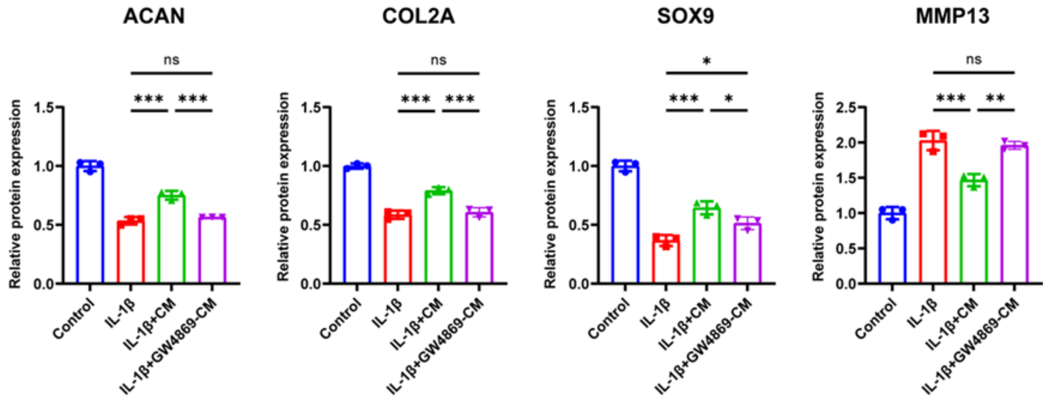

Supplementary Figure 2. Protein expression levels after GW4869 treatment. Western blot quantitative analysis reveals that after the addition of GW4869, the expression levels of SOX9, ACAN, and COL2A are not significantly different from those in the model group, and MMP13 expression is not reduced. Statistical significance was assessed using one-way ANOVA for multiple group comparisons, followed by post hoc tests. All data are presented as mean ± SD (n=3). *p < 0.05, **p < 0.01, *p < 0.001, and ns = no statistically significant difference between groups

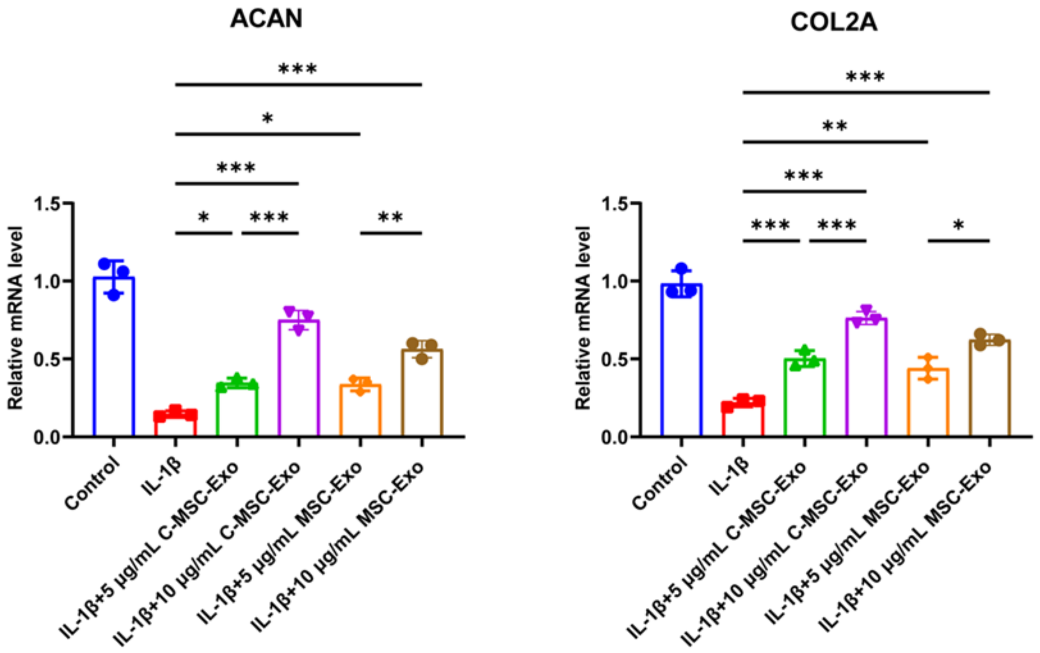

Supplementary Figure 4. Expression of the chondrogenic-related genes ACAN and COL2A increases after the addition of CyA-MSC-derived exosomes, demonstrating a dose-dependent relationship. Statistical significance was assessed using one-way ANOVA for multiple group comparisons, followed by post hoc tests. All data are presented as mean ± SD (n=3). *p < 0.05, **p < 0.01, *p < 0.001, and ns = no statistically significant difference between groups.

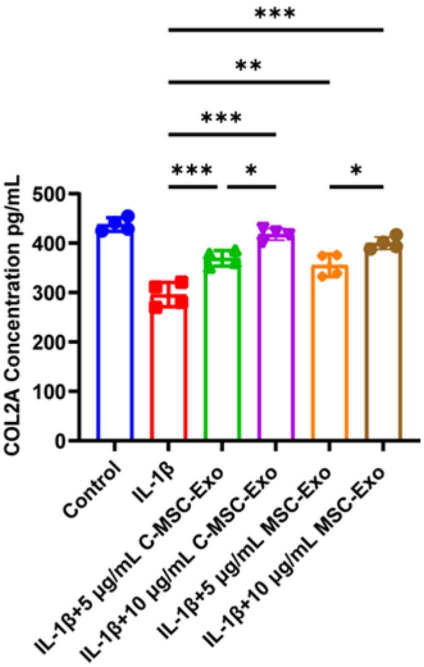

Supplementary Figure 5. ELISA detection reveals that COL2A expression increases after the addition of CyA-MSC-derived exosomes. Statistical significance was assessed using one-way ANOVA for multiple group comparisons, followed by post hoc tests. All data are presented as mean ± SD (n=3). *p < 0.05, **p < 0.01, and *p < 0.001.

© 2025. An X et al. Published online at http://www.aginganddisease.org/EN/10.14336/AD.2024.10016

SUPPLEMENTARY DATA

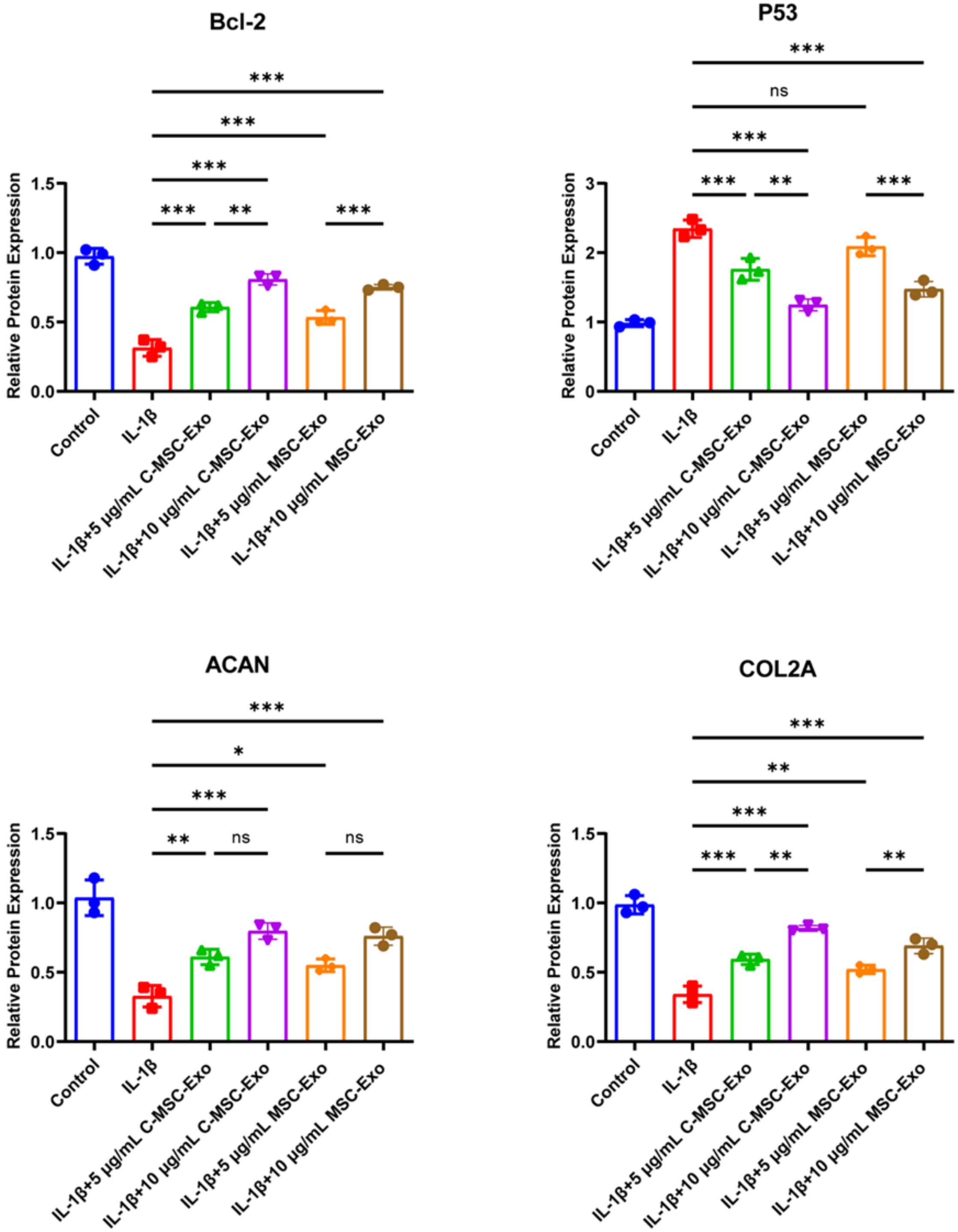

Supplementary Figure 6. Western blot quantitative analysis reveals that after the addition of CyA-MSC-derived exosomes, the expression levels of ACAN, COL2A, C-Myc, and Bcl-2 increase, while the expression levels of IKK-α, IKK-β, P50, P53, and P56 decrease. Statistical significance was assessed using one-way ANOVA for multiple group comparisons, followed by post hoc tests. All data are presented as mean ± SD (n=3). *$p < 0.05$, **$p < 0.01$, *$p < 0.001$, and ns = no statistically significant difference between groups.

© 2025. An X et al. Published online at http://www.aginganddisease.org/EN/10.14336/AD.2024.10016

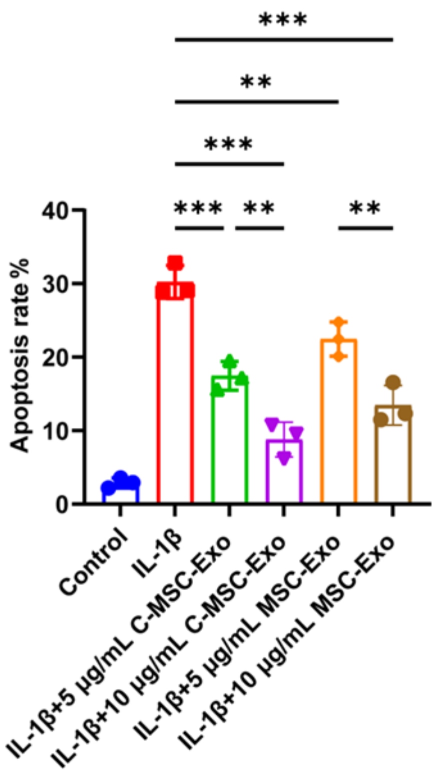

Supplementary Figure 7. Quantification of Apoptosis. Quantitative analysis reveals that cell apoptosis decreases following the addition of CyA-MSC-derived exosomes. Statistical significance was assessed using one-way ANOVA for multiple group comparisons, followed by post hoc tests. All data are presented as mean ± SD (n=3). **p < 0.01 and *p < 0.001.

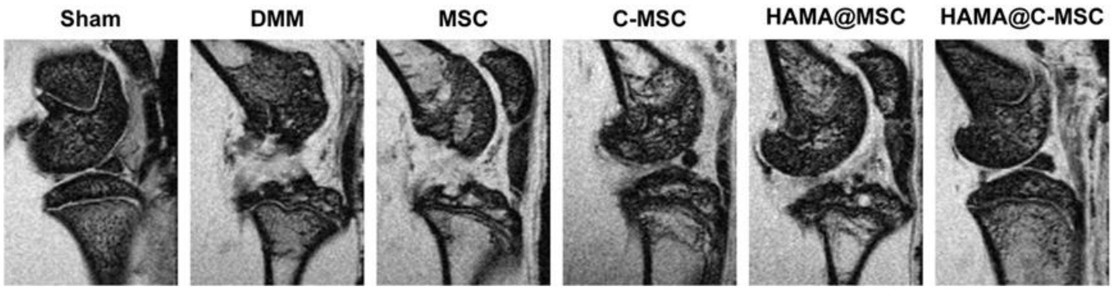

Supplementary Figure 8. MRI results of DMM rat knee joints.

© 2025. An X et al. Published online at http://www.aginganddisease.org/EN/10.14336/AD.2024.10016

SUPPLEMENTARY DATA

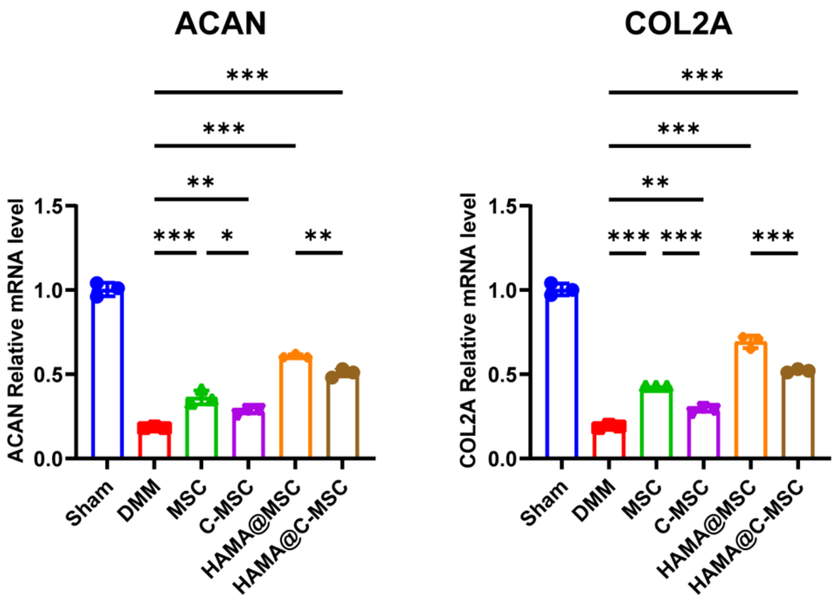

Supplementary Figure 9. Relative mRNA Expression of ACAN and COL2A in Joint Tissues Across Different Groups. Statistical significance was assessed using one-way ANOVA for multiple group comparisons, followed by post hoc tests. All data are presented as mean ± SD (n=3). *p < 0.05, **p < 0.01, and *p < 0.001.

© 2025. An X et al. Published online at http://www.aginganddisease.org/EN/10.14336/AD.2024.10016
